# Hypothalamic-mediated control of glucose balance in the presence and absence of insulin

**DOI:** 10.18632/aging.100641

**Published:** 2014-03-02

**Authors:** Teppei Fujikawa, Roberto Coppari

**Affiliations:** ^1^ Department of Internal Medicine, Division of Hypothalamic Research, The University of Texas Southwestern Medical Center, Dallas, TX 75390, USA; ^2^ Department of Cellular Physiology and Metabolism, University of Geneva, Geneva 1211, Switzerland

**Keywords:** Diabetes, leptin, insulin, hypothalamus

## Abstract

Diabetes afflicts hundreds of millions worldwide. People affected by type 1 diabetes mellitus (T1DM; the insulin-deficient form of diabetes) or type 2 diabetes mellitus (T2DM; the insulin-resistant form of diabetes) have significantly reduced life expectancy compared to normal individuals. This is due in part to the fact that (despite improvements) current anti-diabetic approaches are suboptimal. Indeed, severe morbidities (e.g.: cardiovascular disease, hypertension) are still too often associated with diabetes. Recent preclinical results indicate that different types of hypothalamic neurons are endowed with the ability to mediate the hyperglycemia-lowering action of the adipocyte-derived hormone leptin in an insulin-dependent and insulin-independent fashion. These results may pave the way for better anti-diabetic approaches and therefore positively impact on life expectancy of diabetic subjects.

“Don't be trapped by dogma — which is living with the results of other people's thinking.” – Steve Jobs, 2005

## Glucose homeostasis and Leptin

The dogma that the pancreatic-beta-cell-secreted hormone insulin is an absolute prerequisite for survival has withstood for almost a century. Precisely, since the early 1920s when insulin was first discovered and shown to rescue the lethality and part of the metabolic aberrancies caused by insulin deficiency in humans [1]. However, recent results from different groups have called into question the notion that life without insulin is incompatible. In 2008, Unger and colleagues reported that leptin overexpression is capable of rescuing the sever hyperglycemia and lethality brought on by insulin deficiency in rodents [2]. These effects are not mediated by direct action of leptin on hepatocytes [3] or pancreatic alpha cells (that are specialized in the secretion of the glycemia-rising hormone glucagon)[4, 5]. Rather, these anti-diabetic and lifesaving effects are due to the action of leptin on brain neurons [4, 6]. Specifically, leptin acts on neurons endowed with the ability to express leptin receptor and also vesicular γ-aminobutyric acid (GABA) transporter (VGAT) that biochemically characterizes GABAergic neurons [7, 8]. A small but important contribution is also made by direct action of leptin on hypothalamic proopiomelanocortin (POMC) neurons that belong to the central melanocortin system [9]. These results indicate that an intricate hypothalamic neurocircuitry is empowered with the ability to exert anti-diabetic actions independently to insulin.

Historically, the anti-diabetic property of leptin was first shown in 1995 when results from studies aimed at testing the effect of leptin administration in insulin resistant rodents were made available [10-12]. Notably, the anti-diabetic effect of leptin was shown to be independent to its well-established body-weight-reducing and food-intake-suppressing actions. In fact, systemic leptin administration at a dose that does not affect body weight and food intake was shown to ameliorate hyperglycemia in *ob/ob* mice [these mice are characterized by a mutation in the gene encoding for leptin (*ob* gene) and are leptin deficient] [10].

**Figure 1:**
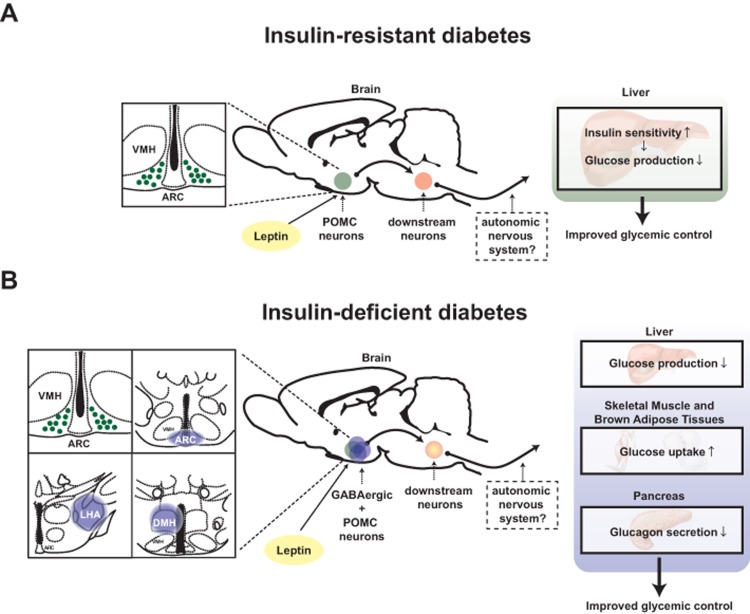
Distinct mechanisms underlie the anti-diabetic actions of leptin in the context of insulin deficiency versus insulin resistance Schematic figures depicting pathways by which leptin exerts anti-diabetic actions: (**A**) leptin acts on leptin receptors in hypothalamic proopiomelanocortin (POMC) neurons to regulate hepatic insulin sensitivity hence improve glucose levels in the blood of T2DM mice [17]. (**B**) leptin acts on leptin receptors in hypothalamic POMC and γ-aminobutyric acid (GABA)ergic neurons to regulate liver, interscapular brown adipose tissue and soleus muscle function to reduce glucose levels in the blood of T1DM mice [4]. LHA; lateral hypothalamic area, ARC; hypothalamic arcuate nucleus, DMH; dorsomedial nucleus and VMH; ventromedial hypothalamic nucleus. Anatomical location of LHA, ARC and DMH is shown in blue-colored circle [48]. POMC neurons are depicted in green while GABAergic neurons in blue.

A large body of evidence indicates that hypothalamus is a crucial site in which changes in circulating hormones and metabolites level are first detected and where this information is processed and then conveyed to downstream pathways aimed at maintaining normal energy and glucose homeostasis [13]. The glycemia-lowering effect of leptin appears to function *via* direct action of the hormone on to this brain region also. For example, microinjection of leptin into the ventromedial hypothalamic nucleus (VMH) enhances glucose uptake in skeletal muscle, brown adipose tissues and other organs [14]. Also, adenovirally-mediated re-expression of leptin receptors (LEPRs) only in the hypothalamic arcuate nucleus (ARC) was shown to be sufficient to ameliorate hyperglycemia in otherwise LEPR null mice [15]. As a follow up to the aforementioned study, Huo and colleagues demonstrated that overexpression of LEPR-B (a biologically functional isoform of leptin receptor) only in POMC neurons is sufficient to ameliorate hyperglycemia in otherwise LEPRs null mice [16]. In line with this study, Berglund and colleagues confirmed that physiological re-expression of LEPRs only in POMC neurons in the ARC brings about normalization of the hyperglycemia caused by an otherwise complete LEPRs deficiency [17]. Moreover, Berglund and colleagues showed that the glycemia-lowering action of leptin *via* action on POMC neurons is due, in part, to increased insulin sensitivity by the liver [17] (Figure 1).

The aforementioned studies were performed in mice able to produce insulin. Therefore, they were not able to determine whether insulin is a prerequisite for the glycemia-lowering action of leptin. Further elucidating the role of insulin on this pathway, our recently published results indicate that the anti-diabetic effect of leptin mediated by POMC neurons requires the presence of insulin [4]. In fact, we reported that physiological re-expression of LEPRs only in POMC neurons in the ARC is not sufficient to bring about the anti-diabetic action of leptin administration in the context of insulin deficiency [4]. Collectively, these results indicate that depending on whether insulin is present or absent different hypothalamic neurons are crucially involved in mediating the glycemia-lowering action of the hormone. Precisely, in the insulin-resistant state the vast majority of the anti-diabetic action of leptin is mediated by POMC neurons whereas in the insulin-deficient state this effect is mainly mediated by GABAergic neurons [4, 16, 17] (Figure 1).

## Leptin, Hypothalamus and Aging

Results from genetic studies recently indicated that manipulation of genes restrictedly in specific hypothalamic nuclei could accelerate or decelerate aging process in mice [18-21]. For example, inflammation signal is slightly and gradually increased during aging, and this low-grade inflammation is thought to be among the factors promoting whole-body aging process [22]. Zhang and colleagues showed that genetically-induced low-grade inflammation in the hypothalamus shortens the lifespan in mice whereas inhibition of hypothalamic low-grade inflammation extends it [19]. Sirtuins are a class of nicotinamide adenine dinucleotide (NAD^+^)-dependent enzymes that mediate a variety of biological function and play a key role in the regulation of aging process [23]. Of note, SIRT1 (a mammalian ortholog of yeast Sir2) is highly expressed by hypothalamic neurons [24] and deletion of SIRT1 from POMC neurons accelerates the age-dependent decline in brown adipocyte mass in perigonadal fat [21, 23]. It is known that skeletal muscle insulin sensitivity declines with aging [25]. Interestingly, SIRT1 in steroidogenic factor 1 (SF1)-expressing neurons (that are found only in the VMH) antagonizes the age-dependent decline in insulin sensitivity in skeletal muscle [20]. Moreover, in 2013, Satoh and colleagues found that brain-restricted overexpression of SIRT1 extends lifespan in mice [18]. They speculated that this anti-aging effect of SIRT1 overexpression is due to enhanced SIRT1 action in two hypothalamic sites, namely the dorsomedial nucleus and later hypothalamic area (DMH and LHA, respectively) [18]. Although future studies aimed at pinpointing the precise contribution of SIRT1 overexpression in different hypothalamic nuclei on lifespan are needed, these results further bolster the idea that hypothalamus exerts a crucial role on regulating the pace of aging and the lifespan of a mammalian organism. Thus, approaches aimed at maintaining the hypothalamus “young” should lead to increased lifespan. On the contrary, maneuvers aimed at speed up the aging process in the hypothalamus (e.g.: feeding on fat-rich foods) should lead to reduced lifespan.

The aforementioned results somewhat called into question the idea that aging process is programmed in each cell and regulated in a cell-autonomous manner. Instead, these results would indicate that the hypothalamus regulates systemic aging and an organism lifespan; a notion that was first proposed by Dilman approximately 60 years ago [26]. It is safe to say that the concept that neurons regulate lifespan is well-appreciated in other species as for example the worm *Caenorhabditis elegans* and the fly *Drosophila melanogaster* [27, 28]. Genetic manipulation of the insulin signaling in neurons extends the lifespan up to two or three folds in worms [29, 30]. Thus, it seems as neurons can govern aging process in an insulin-depend manner [30]. Obviously, maintaining normal energy homeostasis is crucial for normal aging as it is demonstrated that increased energy intake shortens, while calorie restriction lengthens, lifespan in many organisms, including non-human primates [31-33]. As mentioned above, by integrating hormonal signals (e.g.: leptin) into coordinated outputs the hypothalamus governs energy homeostasis and several metabolic pathways in peripheral tissues [13]. Because SIRT1 in POMC and SF1 neurons has been shown to regulate different parameters of the aging process, in retrospect, the idea that hypothalamic neurons governs longevity does not appear to be bizarre. Nevertheless, this notion has currently earned support from solid scientific results [18, 19].

Leptin may be involved in the aging process, as systemic deficiency of either leptin or its receptor causes shorted lifespan [34, 35]. However, the mechanism by which leptin directly contributes to aging process is far less understood compared to the modality by which insulin affects this parameter. The insulin receptor signaling pathway is conserved from worms to humans, and numerous studies have shown that decreased insulin signaling leads to longer lifespan while increased insulin signaling does exactly the opposite. For instance, reactivation of *daf-2* or *age-1* (which are insulin signaling pathway genes in worm) only in neurons can abrogate the increased lifespan phenotype brought on by an otherwise whole-body *daf-2* or *age-1* ablation [30]. Also, genetic inhibition of insulin receptor signaling in the brain results in extended lifespan in rodents [36, 37]. These results suggest that the mechanism by which insulin signaling pathway regulates lifespan is conserved from worms to mammals.

Contrary to insulin, leptin is found in vertebrates and no obvious orthologs in lower specie have been described yet [38]. Rajan and Perrimon recently reported that the fly *Drosophila melanogaster* has a leptin-like hormone, namely cytokine unpaired 2 (*Upd2*). However, the physiological function of *Upd2* appears to be opposite to the one described for leptin [39]. Thus, it is unclear whether leptin and leptin-like hormone share similar functions. As mentioned above, systemic deficiency of either leptin or its receptors shortens lifespan [34, 35]. However, it is not well-defined whether the shortened lifespan results directly from lack of leptin signaling or to the long array of metabolic defects engendered by its deficiency. In fact, lack of leptin signaling causes obesity, diabetes and other aberrancies that promote an aging process and are expected to shorten lifespan. Nevertheless, human studies demonstrated that circulating leptin levels decrease with aging independently to fat mass and other age-related endocrine changes [40]. Also, rodents studies showed that leptin resistance in aged animals occurs independently to changes in fat mass [41], suggesting that leptin actions in the brain is likely attenuated during aging. Two studies have indicated that leptin is important for neuronal development and more specifically for normal neuronal projections and plasticity [42, 43]. Leptin level is dramatically increased in the early postnatal stage [44]. *ob/ob* mice have structural neuronal abnormalities [42, 45, 46]. Bouret and colleagues showed that leptin treatment rescues these aberrant neuronal projections at early postnatal age [42]. Also, Pinto and colleagues demonstrated that leptin could rapidly induce synaptic rewiring of hypothalamus of adult mice; in fact, they showed that leptin injection into *ob/ob* mice increases synaptic inputs to anorexigenic POMC neurons and decreases these inputs to orexigenic neurons (e.g.: Agouti-related protein/neuropeptide Y neurons) [43]. Thus, by affecting wiring and function of specific hypothalamic neurons leptin may directly be involved in the regulation of an organism lifespan.

## Are Novel Anti-diabetic Approaches in the Horizon?

Age-related maladies include diabetes, a defect that increases the risk of other complex diseases including cardiovascular disease (one of the leading cause of death worldwide) [9]. Diabetes is characterized by altered glucose metabolism causing increased circulating level of glucose. It has been suggested that an excess in energy substrate (e.g.: glucose) increases intracellular oxidative damage and hence promotes cellular aging process [47]. Thus, approaches aimed at reducing circulating glucose are expected to improve lifespan of diabetic subjects. Recent results indicate that hypothalamic neurons are empowered with the ability to suppress diabetic hyperglycemia in an insulin-independent fashion. These findings are expected to lead to better anti-diabetic approach. For example, these results indicate in LEPRs-expressing GABAergic neurons (that are found only in LHA, DMH and ARC) as crucial mediators of the anti-diabetic action of leptin in the context of insulin deficiency [4]. Further studies are however needed to identify i) which of the neurons within the LHA, DMH, and ARC are really crucial (one likely candidate is AgRP neurons that are found in ARC), ii) which neurons are downstream to these first-order GABAergic neurons, iii) what are the peripheral effectors underlying the effects of leptin in insulin deficiency. Obviously if these studies were to identify a molecule able to suppress hyperglycemia in an insulin-independent fashion, then, this molecule has the potential to lower hyperglycemia in both forms of diabetes and lengthen lifespan of diabetic people. These studies are ongoing and time will tell whether harnessing of a hypothalamic-dependent circuitry can suppress hyperglycemia and hence extend lifespan of humans affects by T1DM and T2DM.
